# Knowledge, Attitude, and Practice in Dental Trauma Management Among Schoolteachers in Taif, Saudi Arabia

**DOI:** 10.3390/healthcare13020200

**Published:** 2025-01-20

**Authors:** Muaath H. Alzahrani, Muwffak Alghoraibi, Mohammed A. Alzubaidi, Sakeenabi Basha, Yousef Althomali, Roshan Noor Mohamed, Faisal K. Altalhi, Yazeed A. Alzahrani, Amal Albalooshy, Abdulaziz Alharbi, Ali Alqarni

**Affiliations:** 1 Independent Researcher, Taif 26526, Saudi Arabia; muaath17@tudent.org; 2 Independent Researcher, Taif 26314, Saudi Arabia; mowaffak54@tudent.org; 3 Department of Preventive Dentistry, Faculty of Dentistry, Taif University, Taif 21944, Saudi Arabiasakeena@tudent.org (S.B.); yalthomali@tudent.org (Y.A.); roshan.noor@tudent.org (R.N.M.); amal.m@tudent.org (A.A.); 4 Faculty of Dentistry, Taif University, Taif 21944, Saudi Arabia; faisalk1144@tudent.org (F.K.A.); yazyd61@tudent.org (Y.A.A.); 5 Department of Oral and Maxillofacial Surgery and Diagnostic Sciences, Faculty of Dentistry, Taif University, Taif 21944, Saudi Arabia; a.alharbi@tudent.org

**Keywords:** dental, trauma, management, KAP, teachers, Taif

## Abstract

**Background:** In Saudi Arabia, dental trauma is regarded as one of the most important issues affecting schoolchildren. This study evaluated Saudi Arabian schoolteachers’ knowledge, attitude, and practice regarding emergency dental trauma management. **Methods:** A cross-sectional study was conducted on 263 schoolteachers from 25 schools; 50.9% were females. A questionnaire was used to collect respondents’ personal and professional data and information on managing dental injury scenarios and the respondents’ attitudes toward dental injuries. A multivariate logistic regression analysis was carried out for the independent and outcome variable of inadequate knowledge regarding dental trauma management. **Results:** Male teachers had better knowledge than female teachers regarding emergency management of avulsed teeth. Compared to female teachers, male teachers would scrub a tooth with a toothbrush to replant it back into its socket if it fell onto the ground and was covered with dirt. Males had a significantly higher percentage of believing it is not teachers’ responsibility to care for tooth injuries in schools. Teachers with teaching experience of 5–10 years had a significantly higher percentage of training in dental emergencies. Teachers with bachelor’s degrees agreed on the need for teacher involvement to save the tooth in a timely manner. Compared to general teachers, physical education teachers would put the tooth back in the mouth and send the child to the dentist immediately if they were hit in the face and the upper front tooth fell out of their mouth. Teachers with a diploma level of education and teachers with a general type of teaching qualification were 2.15 times (CI = 0.98–3.11, *p* = 0.002) and 3.19 times (CI = 1.71–4.22, *p* = 0.0001) more likely to have a higher level of inadequate knowledge regarding dental trauma management. **Conclusions:** There is a need to raise teachers’ awareness and improve their dental trauma emergency management training.

## 1. Introduction

Dental trauma is considered a public health problem due to its frequency, associated complications, and impact on quality of life [1]. It has long-lasting emotional and social repercussions in addition to immediate medical, cosmetic, and psychological impacts [1,2]. The prevalence of dental trauma varies between countries. Systematic reviews have shown an overall pooled prevalence of dental trauma in permanent teeth ranging from 13% [3] to 19.48% [4] and 24.2% in primary teeth [5]. There is an annual incidence rate of 2.82% (95 CI, 2.28–3.42%) per 100 person-years [6]. It manifests as injury to enamel, dentin, or pulp, dislodged teeth, crush injuries, or tooth loss [1]. Maxillary incisors are the most commonly avulsed teeth in both primary and permanent dentition [7]. The maxillary anterior contributes to esthetics and plays an important role in phonetics, the integrity of supporting tissues, and children’s mental and psychological well-being [1,7].

In Saudi Arabia, dental trauma is considered a significant problem that impacts schoolchildren. Majed et al. conducted a study in which they evaluated the prevalence of dental trauma in boys aged 5–6 and 12–14 in Riyadh, Saudi Arabia. They discovered that 34% of schoolboys had dental trauma [8]. Furthermore, according to a study by Al-Malik conducted in Jeddah, Saudi Arabia, which looked at oral injuries in children visiting a hospital, 9- to 11-year-olds had the highest rate of dental injuries [9]. Similarly, Al-Ansari’s study in Dammam/Al-Khobar, Saudi Arabia, assessed the dental trauma prevalence among schoolboys in the Eastern Province of Saudi Arabia, finding that 39.5% of the boys had experienced dental trauma [10].

Providing prompt and adequate emergency treatment and counseling is critical to the prognosis of dental trauma and is usually the responsibility of qualified personnel at the accident site [10,11]. It is imperative to provide emergency care that is both high-quality and timely in order to prevent negative outcomes [11,12,13].

Children sustain oral injuries primarily at home, with schools being the second most prevalent site [1]. A study conducted by Fittler A. et al. [14] in Hungary showed that more than half of the teachers previously witnessed dental trauma; however, teachers’ knowledge of dental trauma management was inadequate. A study assessing sports teachers’ knowledge of oral–facial trauma occurrence and prevention in the southern region of Saudi Arabia revealed that 88% of the teachers had observed orofacial trauma in students participating in school sports [11]. Several studies have been conducted internationally [15,16,17,18,19,20,21,22,23,24] and nationally [25,26,27,28,29] to evaluate schoolteachers’ knowledge of managing dental trauma. However, previous studies [15,16,17,18,19,20,21,22,23,24,25,26,27,28,29] have revealed a lack of adequate knowledge among schoolteachers regarding dental trauma management. A recent systematic review conducted by Tewari N et al. [30] showed that schoolteachers had low self-belief and knowledge levels regarding dental trauma management, highlighting the need for well-designed questionnaire studies. Most children spend about 40% of their awake time at school [16]. Since sports and school injuries account for about 50% to 88% of overall causes of dental trauma [11,14] and teachers are generally present at the time when dental injuries occur during school hours [16], the prognosis of injured teeth depends on prompt action and appropriate treatment, which often relies on the knowledge of schoolteachers, especially those who teach sports. The present study evaluated schoolteachers’ knowledge, attitudes, and practices (KAP) concerning emergency dental trauma management in Taif, Saudi Arabia.

## 2. Materials and Methods

### 2.1. Study Design, Location, and Time

The present cross-sectional survey was conducted across three months in Taif, Saudi Arabia, from 15 May 2023 to July 2023.

### 2.2. Study Participants

The inclusion criteria were teachers of both genders who had agreed to participate in the study and taught at primary, middle, and secondary schools. The exclusion criteria were kindergarten teachers who refused to participate in the study.

### 2.3. Sampling Methodology

A pilot study was conducted among 25 schoolteachers (pilot study sample not included in final study sample). Based on the pilot study’s results, the sample size of 245 was calculated with a precision of 20% and an error of 5%. The final sample size was rounded to 270 to compensate for non-response bias. A total of 25 schools (5 schools from each region of Taif City) were randomly selected for the study. A total of 263 teachers (129 male and 134 female) agreed to participate and signed the written informed consent for the study.

### 2.4. Ethical Considerations

Ethical approval was obtained from the Institutional Review Committee before starting this study (ethical clearance number: TU-44-324).

### 2.5. Data Collection

A pretested questionnaire was written in English, translated into Arabic, and returned to English for validation. The questionnaire reliability was tested with a Cronbach alpha value of 0.81. There were three sections to the questionnaire.

Part 1 of the questionnaire asked about the respondents’ personal and professional profiles, including age, gender, educational level, professional experience, type of teaching, first aid training, dental emergency training, and previous experience with dental trauma.

Part 2 asked about the urgent management of three scenarios involving dental injury (Case I: a 9-year-old child fell and broke her upper front tooth; Case II: a 12-year-old boy was hit in the face and his upper front tooth fell out of his mouth).

Part 3 used a 5-point Likert scale to describe the respondents’ attitudes toward dental injuries [31].

### 2.6. Statistical Analysis

The collected data were organized and analyzed using the Statistical Package for Social Science (SPSS, Version 27, IBM Corp. 2020 Chicago, IL, USA: SPSS Inc.). The difference in proportion was tested using the Chi-square test. Multivariate logistic regression analysis was carried out for the independent variables (gender, educational level, professional experience, first aid training, witnessing of dental trauma at school, and type of teaching) and the outcome variable of inadequate knowledge regarding dental trauma management. The significance level was set at a *p*-value less than 0.05.

## 3. Results

Table 1 shows the distribution of dental trauma knowledge, attitude, and practice according to gender. The results showed that male teachers have better knowledge than female teachers regarding the emergency management of avulsed teeth. When asked what they would do if they decided to replant a tooth back into its socket but it had fallen onto the ground and was covered in dirt, 21.7% of male teachers and 9% of female teachers agreed that they would scrub the tooth gently with a toothbrush. The difference was statistically significant (*p* = 0.042). When they were asked if it is the moral responsibility of teachers to take care of tooth injuries during school hours, 56.6% of males believed that it is not the teacher’s responsibility compared to females (37.3%). The difference was statistically significant (*p* = 0.041).

Individuals across various educational levels, including bachelor’s degree holders, agree when asked about receiving comprehensive treatment from professionals. Teachers need to be involved in saving the tooth promptly; 24.3% of the study sample held bachelor’s degrees, and 25% held other educational levels. The difference was statistically significant (*p* = 0.041) (Table 2).

Table 3 shows that an increase in teachers’ years of experience correlates with a higher incidence of trauma cases, with the difference being statistically significant (*p* = 0.032).

When inquiring about a 12-year-old boy who was hit in the face, resulting in the loss of an upper front tooth, 37.5% of physical education teachers indicated that their immediate emergency action would be to put the tooth back in its place in the mouth and send the child to the dentist immediately. This percentage is notably higher than general teachers, with the difference being statistically significant (*p*= 0.042) (Table 4).

Table 5 shows a multivariate logistic regression model of factors associated with the teachers’ inadequate knowledge regarding dental trauma management. Teachers with less than 5 years of professional experience had a 1.17 times (CI = 0.1–2.13, *p* = 0.041) greater level of inadequate knowledge regarding dental trauma management. Teachers with a diploma had a 2.15 times (CI = 0.98–3.11, *p* = 0.002) greater level of inadequate knowledge regarding dental trauma management. General teachers had a 3.19 times (CI = 1.71–4.22, *p* = 0.0001) greater level of inadequate knowledge regarding dental trauma management compared to physical education teachers. Teachers who did not witness dental trauma had a 1.18 times (CI = 0.13–2.15 *p* = 0.004) greater level of inadequate knowledge regarding dental trauma management.

## 4. Discussion

To the best of the authors’ knowledge, this is the first study from the western province of Saudi Arabia to assess the emergency care of traumatic dental injuries (TDIs) in schools and the knowledge, attitudes, and practices of schoolteachers. Given the age of the children and the amount of time they spend in school, it is reasonable to assume that teachers will encounter oral trauma scenarios regularly [25].

Comparable to Al-Khalifa et al.’s study in the Dammam region of Saudi Arabia [26], the current study’s results indicate that 65.1% of male instructors and 65.7% of female instructors saw dental trauma among students in their classrooms. This is, nevertheless, significantly greater than in studies carried out in Al Madinah (6.2%) [25] and Riyadh (23%) [27], Saudi Arabia, and Samsun (40%), Turkey [21]. This may be due to unsupervised sporting events performed on school property; dangerous school circumstances may also be associated with traumatic dental injuries occurring on school premises [21,25].

In the present study, only 21.7% of male instructors and 22.4% of female instructors reported taking oral injuries courses. Among those teachers, 22% received training in dental emergency management, with only 32.6% of male teachers and 25.4% of female teachers obtaining first aid training, exacerbating the problem due to a general lack of knowledge in dental first aid protocols. This level of training is lower than that observed in a previous study conducted in Madinah, where 28.1% had acquired first aid training in trauma [25]. However, schoolteachers’ knowledge and attitudes toward treating broken upper front and avulsed teeth were similar to those reported in other studies [25,27,28].

Nonetheless, only a small percentage of educators were trained in dental emergencies; 86% of male and 92% of female educators properly responded when asked about immediate emergency management for a shattered upper front tooth. When asked how they would proceed if they were to replace a tooth that had fallen out of its socket and was covered in dirt, 93% of teachers gave the following answer: use a toothbrush to gently scrub the tooth and then rinse it with tap water, an antiseptic solution, or alcohol. This exceeded the percentages identified in studies from other nations [15,16,17,18] and various parts of Saudi Arabia, which ranged from 30% to 79% [26,28,29].

When teachers were questioned on the proper storage method for an avulsed tooth, nearly three-fourths chose the correct answer, far surpassing findings from international [15,16,17,18,19,20,22] and local studies [25,29].

In the present study, 45% of male and 52% of female teachers agreed that wearing a mouthguard should be compulsory in all outdoor sports. A total of 58% of male and 64% of female teachers agreed that time is important in saving a tooth during dental injuries. This result is in comparison to international surveys conducted using similar questions [16,23].

In accordance with the previous study conducted by Feldens et al. [24], the present study demonstrated that teachers with a diploma had a 2.15 times higher level of inadequate knowledge regarding dental trauma management compared to teachers with bachelor’s and other degrees, which indicates that the level of education has a positive impact on the teachers’ knowledge about topics that do not belong to their specific area of study, including dental trauma. The teachers with less than 5 years of professional experience had a 1.17 times higher level of inadequate knowledge regarding dental trauma management than teachers with more professional experience. Professional experience, translated into years of work as a teacher, contributes to the teachers’ knowledge about dental trauma [24].

In the present study, the teachers who did not witness cases of trauma had inadequate knowledge compared to teachers who had witnessed dental trauma, suggesting that experiencing situations that involve dental trauma makes teachers more capable of managing these cases properly [24]. The physical education teachers had good knowledge about dental trauma management compared to general teachers, indicating that the education teachers receive during their training positively impacts managing traumatic dental injuries.

The present study showed no significant association between the age of teachers and knowledge of dental trauma management. In contrast, Awad, MA. et al. [32] showed that many younger teachers expressed the need for further education in dental trauma management.

### Study Limitations

The limitations of the present study include the utilization of a self-administered questionnaire, which could introduce recollection bias, as well as the disparity in response rates across different categories; additionally, detailed information about training courses for dental injuries was not included.

## 5. Conclusions

According to this study, male teachers were more knowledgeable than female teachers regarding handling avulsed teeth in emergencies. Instructors with five to ten years of experience in the classroom demonstrated a significantly greater percentage of training in dental emergencies, and those with a bachelor’s degree agreed more strongly that teachers must be involved in saving teeth in a timely manner. Compared to general teachers, physical education teachers demonstrated superior knowledge regarding dental trauma management. It is necessary to increase instructors’ understanding of oral trauma emergency management and to enhance their training in this area. In addition, there is a need for further research in this area, including the types of dental trauma courses taken and whether the courses were required or optional. In addition, more research is required to determine the etiology of the greater incidence of dental trauma in schools in Saudi Arabia’s western province.

## Figures and Tables

**Table 1:** Distribution of dental trauma knowledge, attitude, and practice according to gender.

Questions		Gender		Chi-Square Value
		Male (*n* = 129)	Female (*n* = 134)	
Do you have first aid training?	Yes	42 (32.6)	34 (25.4)	0.125
	No	87 (67.4)	100 (75.6	
Do you have training regarding dental emergencies?	Yes	28 (21.7)	30 (22.4)	0.132
	No	101 (78.3)	104 (77.6)	
How many trauma cases have you seen?	Zero cases	45 (34.9)	46 (34.3)	0.093
	One to two cases	46 (35.7)	51 (38.1)	
	Three to four cases	19 (14.7)	16 (11.9)	
	Five or more cases	19 (14.7)	21 (15.7)	
Case I: A 9-year-old child fell and broke her upper front tooth: would you know if this was a permanent or baby tooth?	Permanent tooth	54 (41.9)	66 (49.3)	0.126
	Baby tooth	53 (41.1)	46 (34.3)	
	Do not know	22 (17.4)	22 (16.4)	
Your immediate emergency management of the case is:	Send the child to the school nurse if available	68 (52.7)	76 (56.7)	0.092
	Contact the parents and advise them to send the child to the dentist immediately	43 (33.3)	47 (35.1)	
	Reassure the child and send her back to class	9 (7)	6 (4.5)	
	Not sure what to do	9 (7)	5 (3.7)	
Case II: A 12-year-old boy was hit in the face, and his upper front tooth fell out of his mouth. Your immediate emergency management of the case is:	Put the tooth back in its place in the mouth and send the child to the dentist immediately	29 (22.5)	29 (21.6)	0.122
	Stop oral bleeding, then send the child home	29 (22.5)	24 (17.9)	
	Put the tooth in a solution and send the child to the dentist immediately	61 (47.3)	65 (48.5)	
	Not sure what to do	10 (7.8)	16 (11.9)	
If you decide to replant a tooth back into its socket, but it had fallen onto the ground and was covered in dirt, what would you do?	Scrub the tooth gently with a toothbrush	28 (21.7)	12 (9)	0.042
	Rinse the tooth under tap water	31 (24)	53 (39.6)	
	Use alcohol to clean the tooth	21 (16.3)	23 (17.2)	
	Use an antiseptic solution to clean the tooth	40 (31.0)	37 (27.6)	
	Put the tooth straight back into the socket without any pretreatment	9 (7.0)	9 (6.7)	
If a liquid is used to transport the tooth, what liquid would you use?	Fresh milk	44 (34.1)	41 (30.6)	0.134
	Child’s mouth	21 (16.3)	19 (14.2)	
	Water	30 (23.3)	31 (23.1)	
	Normal saline	34 (26.4)	43 (32.1)	
It is the moral responsibility of teachers to take care of tooth injuries that happen during school hours.	1	23 (17.8)	17 (12.7)	0.041
	2	17 (13.2)	13 (9.7)	
	3	33 (25.6)	20 (14.9)	
	4	12 (9.3)	19 (14.2)	
	5	44 (34.1)	65 (48.5)	
Time plays an important role in saving a tooth.	1	57 (44.2)	68 (50.7)	0.113
	2	18 (14.0)	18 (13.4)	
	3	28 (21.7)	20 (14.9)	
	4	10 (7.8)	10 (7.5)	
	5	16 (12.4)	18 (13.4)	
When a tooth is lost due to an injury, it can be saved, so treatment is needed.	1	55 (42.6)	67 (50.0)	0.083
	2	29 (22.5)	16 (11.9)	
	3	23 (17.8)	27 (20.1)	
	4	12 (9.3)	13 (9.7)	
	5	10 (7.8)	11 (8.2)	
Management of tooth injuries must be included during teacher training.	1	42 (32.6)	54 (40.3)	0.046
	2	31 (24.0)	8 (6.0)	
	3	35 (27.1)	43 (32.1)	
	4	12 (9.3)	11 (8.2)	
	5	9 (7.0)	18 (13.4)	
Dental trauma management is an emergency situation.	1	64 (49.6)	75 (56.0)	0.041
	2	28 (21.7)	12 (9.0)	
	3	21 (16.3)	28 (20.9)	
	4	11 (8.5)	11 (8.2)	
	5	5 (3.9)	8 (6.0)	
Teacher’s intervention in school dental injuries plays an important role in saving a tooth.	1	60 (46.5)	69 (51.5)	0.032
	2	32 (24.8)	17 (12.7)	
	3	20 (15.5)	24 (17.9)	
	4	11 (8.5)	13 (9.7)	
	5	6 (4.7)	11 (8.2)	
Even though emergency management of tooth injuries is thoroughly taken care of by professionals, teacher involvement is needed to save the tooth.	1	62 (48.1)	68 (50.7)	0.126
	2	30 (23.3)	28 (20.9)	
	3	22 (17.1)	25 (18.7)	
	4	10 (7.8)	5 (3.7)	
	5	5 (3.9)	8 (6.0)	
Wearing a mouthguard should be made compulsory in all outdoor sports.	1	36 (27.9)	46 (34.3)	0.132
	2	23 (17.8)	24 (17.9)	
	3	32 (24.8)	38 (28.4)	
	4	13 (10.1)	12 (9.0)	
	5	25 (19.4)	14 (10.4)	
In emergency situations, no legal considerations will put teachers in trouble.	1	14 (10.9)	12 (9.0)	0.082
	2	12 (9.3)	10 (7.5)	
	3	28 (21.7)	31 (23.1)	
	4	26 (20.2)	19 (14.2)	
	5	49 (38.0)	62 (46.3)	

Data are presented as numbers and percentages; a statistical test was used for analysis at a *p*-value < 0.05; 1 = strongly agree; 2 = agree; 3 = neutral; 4 = disagree; 5 = strongly disagree.

**Table 2:** Distribution of dental trauma knowledge, attitude, and practice according to education level.

Questions		Education Level			Chi-Square Value
		Diploma	Bachelor	Other	
Do you have first aid training?	Yes	17 (31.5)	53 (28.0)	6 (30.0)	0.134
	No	37 (68.5)	136 (72.0)	14 (70.0)	
Do you have training regarding dental emergencies?	Yes	17 (31.5)	38 (20.1)	3 (15.0)	0.083
	No	37 (68.5)	151 (79.9)	17 (85.0)	
How many trauma cases have you seen?	Zero cases	15 (27.8)	70 (37.0)	6 (30.0)	0.113
	One to two cases	23 (42.6)	66 (34.9)	8 (40.0)	
	Three to four cases	8 (14.8)	24 (12.7)	3 (15.0)	
	Five or more cases	8 (14.8)	29 (15.3)	3 (15.0)	
Case I: A 9-year-old child fell and broke her upper front tooth: would you know if this was a permanent or baby tooth?	Permanent tooth	33 (61.1)	75 (39.7)	12 (60.0)	0.112
	Baby tooth	18 (33.3)	77 (40.7)	4 (20.0)	
	Do not know	3 (5.6)	37 (19.6)	4 (20.0)	
Your immediate emergency management of the case is:	Send the child to the school nurse if available	32 (59.3)	99 (52.4)	13 (65.0)	0.124
	Contact the parents and advise them to send the child to the dentist immediately	18 (33.3)	66 (34.9)	6 (30.0)	
	Reassure the child and send her back to class	3 (5.6)	12 (6.3)	0 (0.0)	
	Not sure what to do	1 (1.9)	12 (6.3)	1 (5.0)	
Case II: A 12-year-old boy was hit in the face, and his upper front tooth fell out of his mouth. Your immediate emergency management of the case is:	Put the tooth back in its place in the mouth and send the child to the dentist immediately	14 (25.9)	41(21.7)	3 (15.0)	0.092
	Stop oral bleeding, then send the child home	12 (22.2)	34 (18.0)	7 (35.0)	
	Put the tooth in a solution and send the child to the dentist immediately	23 (42.6)	94 (49.7)	9 (45.0)	
	Not sure what to do	5 (9.3)	20 (10.6)	1 (5.0)	
If you decide to replant a tooth back into its socket, but it had fallen onto the ground and was covered in dirt, what would you do?	Scrub the tooth gently with a toothbrush	11 (20.4)	27 (14.3)	2 (10.0)	0.083
	Rinse the tooth under tap water	15 (27.8)	62 (32.8)	7 (35.0)	
	Use alcohol to clean the tooth	8 (14.8)	35 (18.5)	1 (5.0)	
	Use an antiseptic solution to clean the tooth	20 (37.0)	51 (27.0)	6 (30.0)	
	Put the tooth straight back into the socket without any pretreatment	0 (0.0)	14 (7.4)	4 (20.0)	
If a liquid is used to transport the tooth, what liquid would you use?	Fresh milk	18 (33.3)	62 (32.8)	5 (25.0)	0.081
	Child’s mouth	8 (14.8)	30 (15.9)	2 (10.0)	
	Water	16 (29.6)	40 (21.2)	5 (25.0)	
	Normal saline	12 (22.2)	57 (30.2)	8 (40.0)	
It is the moral responsibility of teachers to take care of tooth injuries that happen during school hours.	1	8 (14.8)	27 (14.3)	5 (25.0)	0.126
	2	9 (16.7)	17 (9.0)	4 (20.0)	
	3	13 (24.1)	39 (20.6)	1 (5.0)	
	4	8 (14.8)	20 (10.6)	3 (15.0)	
	5	16 (29.6)	86 (45.5)	7 (35.0)	
Time plays an important role in saving a tooth.	1	22 (40.7)	95 (50.3)	8 (40.0)	0.063
	2	6 (11.1)	26 (13.8)	4 (20.0)	
	3	13 (24.1)	31 (16.4)	4 (20.0)	
	4	6 (11.1)	13 (6.9)	1 (5.0)	
	5	7 (13.0)	24 (12.7)	3 (15.0)	
When a tooth is lost due to an injury, it can be saved, so treatment is needed.	1	25 (46.3)	85 (45.0)	12 (60.0)	0.057
	2	9 (16.7)	35 (18.5)	1 (5.0)	
	3	7 (13.0)	37 (19.6)	6 (30.0)	
	4	8 (14.8)	16 (8.5)	1 (5.0)	
	5	5 (9.3)	16 (8.5)	0 (0.0)	
Management of tooth injuries must be included during teacher training.	1	23 (42.6)	65 (34.4)	8 (40.0)	0.041
	2	5 (9.3)	31 (16.4)	3 (16.4)	
	3	19 (35.2)	55 (29.1)	4 (20.0)	
	4	5 (9.3)	15 (7.9)	3 (15.0)	
	5	2 (3.7)	23 (12.2)	2 (10.0)	
Dental trauma management is an emergency situation.	1	27 (50.0)	100 (52.9)	12 (60.0)	0.072
	2	9 (16.7)	27 (14.3)	4 (20.0)	
	3	12 (22.2)	34 (18.0)	3 (15.0)	
	4	6 (11.1)	15 (7.9)	1 (5.0)	
	5	0 (0.0)	13 (6.9)	0 (0.0)	
Teacher’s intervention in school dental injuries plays an important role in saving a tooth.	1	31 (57.4)	90 (47.6)	8 (40.0)	0.043
	2	5 (9.3)	41 (21.7)	3 (15.0)	
	3	11 (20.4)	28 (14.8)	5 (25.0)	
	4	5 (9.3)	17 (9.0)	2 (10.0)	
	5	2 (3.7)	13 (6.9)	2 (10.0)	
Even though emergency management of tooth injuries is thoroughly taken care of by professionals, teacher involvement is needed to save the tooth.	1	26 (48.1)	94 (49.7)	10 (50.0)	0.041
	2	7 (13.0)	46 (24.3)	5 (25.0)	
	3	14 (25.9)	29 (15.3)	4 (20.0)	
	4	5 (9.3)	9 (4.8)	1 (5.0)	
	5	2 (3.7)	11 (11)	0 (0.0)	
Wearing a mouthguard should be made compulsory in all outdoor sports.	1	16 (29.6)	60 (31.7)	6 (30.0)	0.121
	2	8 (14.8)	34 (18.0)	5 (25.0)	
	3	15 (27.8)	49 (25.9)	6 (30.0)	
	4	7 (13.0)	17 (9.0)	1 (5.0)	
	5	8 (14.8)	29 (15.3)	2 (10.0)	
In emergency situations, no legal considerations will put teachers in trouble.	1	4 (7.4)	19 (10.1)	3 (15.0)	0.032
	2	1 (1.9)	17 (9.0)	4 (20.0)	
	3	13 (24.1)	42 (22.2)	4 (20.0)	
	4	12 (22.2)	29 (15.3)	4 (20.0)	
	5	24 (44.4)	82 (43.4)	5 (25.0)	

**Table 3:** Distribution of dental trauma knowledge, attitude, and practice according to teachers’ experience.

Questions		School Experience Level			Chi-Square Value
		Less Than 5 Years	5 to 10 Years	More Than 10 Years	
Do you have first aid training?	Yes	13 (33.3)	12 (30.0)	51 (27.7)	0.112
	No	26 (66.7)	28 (70.0)	133 (72.3)	
Do you have training regarding dental emergencies?	Yes	6 (15.4)	12 (30.0)	40 (21.7)	0.041
	No	33 (84.6)	28 (70.0)	144 (78.3)	
How many trauma cases have you seen?	Zero cases	22 (56.4)	15 (37.5)	54 (29.3)	0.032
	One to two cases	12 (30.8)	17 (42.5)	68 (37.0)	
	Three to four cases	3 (7.7)	4 (10.0)	28 (15.2)	
	Five or more cases	2 (5.1)	4 (10.0)	34 (18.5)	
Case I: A 9-year-old child fell and broke her upper front tooth: would you know if this was a permanent or baby tooth?	Permanent tooth	19 (48.7)	12 (30.0)	89 (48.4)	0.074
	Baby tooth	12 (30.8)	17 (42.5)	70 (38.0)	
	Do not know	8 (20.5)	11(27.5)	25 (13.6)	
Your immediate emergency management of the case is:	Send the child to the school nurse if available	19 (48.7)	21 (52.5)	104 (56.5)	0.121
	Contact the parents and advise them to send the child to the dentist immediately	13 (33.3)	11 (27.5)	66 (35.9)	
	Reassure the child and send her back to class	5 (12.8)	5 (12.5)	5 (2.7)	
	Not sure what to do	2 (5.1)	3 (7.5)	9 (4.9)	
Case II: A 12-year-old boy was hit in the face, and his upper front tooth fell out of his mouth. Your immediate emergency management of the case is:	Put the tooth back in its place in the mouth and send the child to the dentist immediately	7 (17.9)	12 (30.0)	39 (21.2)	0.062
	Stop oral bleeding, then send the child home	11 (28.2)	8 (20.0)	34 (18.5)	
	Put the tooth in a solution and send the child to the dentist immediately	19 (48.7)	16 (40.0)	91 (49.5)	
	Not sure what to do	2 (5.1)	4 (10.0)	20 (10.9)	
If you decide to replant a tooth back into its socket, but it had fallen onto the ground and was covered in dirt, what would you do?	Scrub the tooth gently with a toothbrush	6 (15.4)	13 (32.5)	21 (11.4)	0.032
	Rinse the tooth under tap water	12 (30.8)	5 (12.5)	67 (36.4)	
	Use alcohol to clean the tooth	6 (15.4)	8 (20.0)	30 (16.3)	
	Use an antiseptic solution to clean the tooth	11 (28.2)	8 (20.0)	58 (31.5)	
	Put the tooth straight back into the socket without any pretreatment	4 (10.3)	6 (15.0)	8 (4.3)	
If a liquid is used to transport the tooth, what liquid would you use?	Fresh milk	9 (23.1)	17 (42.5)	59 (32.1)	0.042
	Child’s mouth	8(20.5)	6 (15.0)	26 (14.1)	
	Water	9 (23.1)	8 (20.0)	44 (23.9)	
	Normal saline	13 (33.3)	9 (22.5)	55 (29.9)	
It is the moral responsibility of teachers to take care of tooth injuries that happen during school hours.	1	9 (23.1)	6 (15.0)	25 (13.6)	0.033
	2	9 (23.1)	10 (25.0)	11 (6.0)	
	3	2 (5.1)	12 (30.0)	39 (21.2)	
	4	8 (20.5)	4 (10.0)	19 (10.3)	
	5	11 (28.2)	8 (20.0)	90 (48.9)	
Time plays an important role in saving a tooth.	1	18 (46.2)	14 (35.0)	93 (50.5)	0.112
	2	5 (12.8)	8 (20.0)	23 (12.5)	
	3	6 (15.4)	10 (25.0)	32 (17.4)	
	4	7 (17.9)	3 (7.5)	10 (5.4)	
	5	3 (7.7)	5 (12.5)	26 (14.1)	
When a tooth is lost due to an injury, it can be saved, so treatment is needed.	1	11 (28.2)	10 (25.0)	101 (54.9)	0.041
	2	7 (17.9)	16 (40.0)	22 (12.0)	
	3	15 (38.5)	9 (22.5)	26 (14.1)	
	4	5 (12.8)	1 (2.5)	19 (10.3)	
	5	1 (2.6)	4 (10.0)	16 (8.7)	
Management of tooth injuries must be included during teacher training.	1	14 (35.9)	13 (32.5)	69 (37.5)	0.083
	2	8 (20.5)	7 (17.5)	24 (13.0)	
	3	10 (25.6)	11 (27.5)	57 (31.0)	
	4	5 (12.8)	6 (15.0)	12 (6.5)	
	5	2 (5.1)	3 (7.5)	22 (12.0)	
Dental trauma management is an emergency situation.	1	19 (48.7)	18 (45.0)	102 (55.4)	0.113
	2	7 (17.9)	8 (20.0)	25 (13.6)	
	3	8 (20.5)	5 (12.5)	36 (19.6)	
	4	5 (12.8)	5 (12.5)	12 (6.5)	
	5	0 (0.0)	4 (10.0)	9 (4.9)	
Teacher’s intervention in school dental injuries plays an important role in saving a tooth.	1	16 (41.0)	15 (37.5)	98 (53.3)	0.063
	2	7 (17.9)	11 (27.5)	31 (16.8)	
	3	11 (28.2)	10 (25.0)	23 (12.5)	
	4	5 (12.8)	3 (7.5)	16 (8.7)	
	5	0 (0.0)	1 (2.5)	16 (8.7)	
Even though emergency management of tooth injuries is thoroughly taken care of by professionals, teacher involvement is needed to save the tooth.	1	18 (46.2)	14 (35.0)	98 (53.3)	0.032
	2	14 (35.9)	10 (25.0)	34 (18.5)	
	3	6 (15.4)	14 (35.0)	27 (14.7)	
	4	1 (2.6)	1 (2.5)	13 (7.1)	
	5	0 (0.0)	1 (2.5)	12 (6.5)	
Wearing a mouthguard should be made compulsory in all outdoor sports.	1	12 (30.8)	6 (15.0)	64 (34.8)	0.033
	2	6 (15.4)	9 (22.5)	32 (17.4)	
	3	15 (38.5)	12 (30.0)	43 (23.4)	
	4	4 (10.3)	5 (12.5)	16 (8.7)	
	5	2 (5.1)	8 (20.0)	29 (15.8)	
In emergency situations, no legal considerations will put teachers in trouble.	1	5 (12.8)	5 (12.5)	16 (8.7)	0.062
	2	6 (15.4)	4 (10.0)	12 (6.5)	
	3	14 (35.9)	13 (32.5)	32 (17.4)	
	4	8 (20.5)	5 (12.5)	32 (17.4)	
	5	6 (15.4)	13 (32.5)	92 (50.0)	

**Table 4:** Distribution of dental trauma knowledge, attitude, and practice according to teaching type.

Question		Type of Teaching		Chi-Square Value
		Physical Education	General	
Do you have first aid training?	Yes	17 (53.1)	59 (25.5)	0.001
	No	15 (46.9)	172 (74.5)	
Do you have training regarding dental emergencies?	Yes	15 (46.9)	43 (18.6)	0.032
	No	17 (53.1)	188 (81.4)	
How many trauma cases have you seen?	Zero cases	12 (37.5)	79 (34.2)	0.112
	One to two cases	12 (37.5)	85 (36.8)	
	Three to four cases	5 (15.6)	30 (13.0)	
	Five or more cases	3 (9.4)	37 (16.0)	
Case I: A 9-year-old child fell and broke her upper front tooth: would you know if this was a permanent or baby tooth?	Permanent tooth	14 (43.8)	106 (45.9)	0.083
	Baby tooth	15 (46.9)	84 (36.4)	
	Do not know	3 (9.4)	41 (17.7)	
Your immediate emergency management of the case is:	Send the child to the school nurse if available	18 (56.2)	126 (54.5)	0.092
	Contact the parents and advise them to send the child to the dentist immediately	9 (28.1)	81 (35.1)	
	Reassure the child and send her back to class	3 (9.4)	12 (5.2)	
	Not sure what to do	2 (6.2)	12 (5.2)	
Case II: A 12-year-old boy was hit in the face, and his upper front tooth fell out of his mouth. Your immediate emergency management of the case is:	Put the tooth back in its place in the mouth and send the child to the dentist immediately	12 (37.5)	46 (19.9)	0.042
	Stop oral bleeding, then send the child home	7 (21.9)	46 (19.9)	
	Put the tooth in a solution and send the child to the dentist immediately	10 (31.2)	116 (50.2)	
	Not sure what to do	3 (9.4)	23 (10.0)	
If you decide to replant a tooth back into its socket, but it had fallen onto the ground and was covered in dirt, what would you do?	Scrub the tooth gently with a toothbrush	8 (25.0)	32 (13.9)	0.043
	Rinse the tooth under tap water	8 (25.0)	76 (32.9)	
	Use alcohol to clean the tooth	7 (21.9)	37 (16.0)	
	Use an antiseptic solution to clean the tooth	7 (21.9)	70 (30.3)	
	Put the tooth straight back into the socket without any pretreatment	2 (6.2)	16 (6.9)	
If a liquid is used to transport the tooth, what liquid would you use?	Fresh milk	11 (34.4)	74 (32.0)	0.032
	Child’s mouth	8 (25.0)	32 (13.9)	
	Water	6 (18.8)	55 (23.8)	
	Normal saline	7 (21.9)	70 (30.3)	
It is the moral responsibility of teachers to take care of tooth injuries that happen during school hours.	1	7 (21.9)	33 (14.3)	0.041
	2	3 (9.4)	27 (11.7)	
	3	13 (40.6)	40 (17.3)	
	4	3 (9.4)	28 (12.1)	
	5	6 (18.8)	103 (44.6)	
Time plays an important role in saving a tooth.	1	18 (56.2)	107 (46.3)	0.063
	2	3 (9.4)	33 (14.3)	
	3	9 (28.1)	39 (16.9)	
	4	1 (3.1)	19 (8.2)	
	5	1 (3.1)	33 (14.3)	
When a tooth is lost due to an injury, it can be saved, so treatment is needed.	1	11 (34.4)	111 (48.1)	0.031
	2	12 (37.5)	33 (14.3)	
	3	5 (15.6)	45 (19.5)	
	4	1 (3.1)	24 (10.4)	
	5	3 (9.4)	18 (7.8)	
Management of tooth injuries must be included during teacher training.	1	12 (37.5)	84 (36.4)	0.112
	2	8 (25.0)	31 (13.4)	
	3	9 (28.1)	69 (29.9)	
	4	1 (3.1)	22 (9.5)	
	5	2 (6.2)	25 (10.8)	
Dental trauma management is an emergency situation.	1	12 (37.5)	127 (55.0)	0.033
	2	5 (15.6)	35 (15.2)	
	3	11 (34.4)	38 (16.5)	
	4	3 (9.4)	19 (8.2)	
	5	1 (3.10	12 (5.2)	
Teacher’s intervention in school dental injuries plays an important role in saving a tooth.	1	13 (40.6)	116 (50.2)	0.082
	2	6 (18.8)	43 (18.6)	
	3	11 (34.4)	33 (14.3)	
	4	2 (6.2)	22 (9.5)	
	5	0 (0.0)	17 (7.4)	
Even though emergency management of tooth injuries is thoroughly taken care of by professionals, teacher involvement is needed to save the tooth.	1	14 (43.8)	116 (50.2)	0.063
	2	5 (15.6)	53 (22.9)	
	3	9 (28.1)	38 (16.5)	
	4	3 (9.4)	12 (5.2)	
	5	1 (3.1)	12 (5.2)	
Wearing a mouthguard should be made compulsory in all outdoor sports.	1	9 (28.1)	73 (31.6)	0.112
	2	5 (15.6)	42 (18.2)	
	3	10 (31.2)	60 (26.0)	
	4	4 (12.5)	21 (9.1)	
	5	4 (12.5)	35 (15.2)	
In emergency situations, no legal considerations will put teachers in trouble.	1	1 (3.1)	25 (10.8)	0.036
	2	5 (15.6)	17 (7.4)	
	3	8 (25.0)	51 (22.1)	
	4	9 (28.1)	36 (15.6)	
	5	9 (28.1)	102 (44.2)	

Data are presented as numbers and percentages; a statistical test was used for analysis at a *p*-value < 0.05; 1 = strongly agree; 2 = agree; 3 = neutral; 4 = disagree; 5 = strongly disagree.

**Table 5:** Multivariate logistic regression model of factors associated with teachers’ inadequate knowledge regarding dental trauma management.

Variables	Adjusted R^2^	95% CI	*p*-Value
Age in years			
≤35 yr	0.83	0.01–1.92	0.082
>35 yr #			
Gender			
Male #			
Female	1.025	0.07–2.05	0.042
Professional experience			
Less than five years	1.17	0.1–2.13	0.041
Five to ten years	0.96	0.02–1.98	0.073
More than ten years #			
Educational level			
Diploma	2.15	0.98–3.11	0.002
Bachelor or other #			
Teaching type			
Physical education #			
General #	3.19	1.71–4.22	0.0001
Witnessed dental trauma			
Yes #			
No	1.18	0.13–2.15	0.004
Received first aid training			
Yes #	0.86	0.02–2.19	0.09
No			

#: Reference value; CI: confidence interval.

## Data Availability

The raw data supporting the conclusions of this study will be made available by the authors without undue reservation.
